# Effects of Waiting Room and Feline Facial Pheromone Experience on Blood Pressure in Cats

**DOI:** 10.3389/fvets.2021.640751

**Published:** 2021-03-05

**Authors:** Laura R. Van Vertloo, Joyce M. Carnevale, Rebecca L. Parsons, Meghann Rosburg, Suzanne T. Millman

**Affiliations:** ^1^Department of Veterinary Clinical Sciences, Iowa State University, Ames, IA, United States; ^2^Department of Veterinary Diagnostic and Production Animal Medicine, Iowa State University, Ames, IA, United States; ^3^Heartland Animal Hospital, Faribault, MN, United States; ^4^Department of Biomedical Sciences, Iowa State University, Ames, IA, United States

**Keywords:** stress, blood pressure, cats, pheromones, veterinary clinic

## Abstract

Obtaining accurate blood pressure measurements in cats is challenging due to the stressful nature of clinic visits. The objective of this study was to evaluate the effects of veterinary clinic waiting experiences and a feline pheromone spray on blood pressure in the cat. We hypothesized that reduced stress associated with bypassing the waiting room and use of synthetic feline facial pheromone (FFP) spray would result in lower blood pressure. A 2 × 2 factorial design involved two rooms and two FFP treatments. Thirty-nine healthy adult cats were recruited and were systematically assigned to four treatment combinations administered over four visits in 2016 and 2017. Cats were kept in the hospital waiting room or were taken directly to the exam room, with or without FFP treatment. All cats were then acclimated to the exam room for an additional 10 min, where vocalizations were recorded manually, before blood pressure measurements were collected using Doppler ultrasonography. Data were analyzed using generalized linear mixed models, with room × FFP interaction, visit, sex, and trial year in the model. There was no significant effect of waiting room by FFP interaction on blood pressure (*n* = 0.95). Mean blood pressure was significantly higher at visit 1 than visits 2 and 4 (*P* < 0.01), but higher at visit 3 than visit 2 (*n* = 0.02). Mean blood pressure was higher in males (*n* = 0.01), and males were more likely to be categorized as borderline hypertensive/hypertensive or severely hypertensive (*n* = 0.01). Number of vocalizations was significantly associated with waiting room by FFP interactions (*P* < 0.01), with fewer vocalizations associated with bypassing the waiting room and when FFP was provided. In conclusion, although we found some behavioral evidence supporting stress reduction when feline patients bypass the waiting room and are provided with FFP, these interventions did not result in lower blood pressure in a clinical setting.

## Introduction

Systemic hypertension (high blood pressure) is a common complication of diseases seen frequently in aging cats, particularly chronic kidney disease and hyperthyroidism (1–5). The ability to correctly identify cats with hypertension is imperative, because when left untreated hypertension can lead to progression of renal disease, retinal detachment, and damage to the central nervous system and cardiovascular system (6, 7). Conversely, inappropriate treatment due to misdiagnosis can result in iatrogenic hypotension.

Accurate diagnosis and monitoring of hypertension presents particular challenges to feline practitioners, because cats often experience fear and anxiety during veterinary visits (8–11). Feline blood pressure has been shown in some studies to be higher in the hospital environment than the home environment (10, 12), but not in others (11). Concurrently, stress behaviors were associated with higher blood pressure (10, 11), suggesting that stressful situations have the potential to result in increased blood pressure in this species. The concern that stress may artificially increase blood pressure can potentially lead practitioners to dismiss high readings (12, 13).

American Association of Feline Practitioners (AAFP) and International Society of Feline Medicine (ISFM) have developed guidelines for reducing fear and anxiety associated with veterinary visits (14). While there are numerous stressors associated with veterinary visits, and various recommendations to mitigate them (14), we chose to focus specifically on the waiting room and the use of feline facial pheromone (FFP). The veterinary clinic waiting room has been shown to be a source of stress for both feline and canine patients (9, 12, 15, 16), and published guidelines for “feline friendly” practices suggest modifying the waiting room environment or avoiding it entirely (14, 17, 18). Use of FFP is another intervention recommended due to its potential to reduce anxiety-related behaviors in cats (14, 18–20). The impact of FFP and avoiding the waiting room on feline blood pressure have not been studied.

The primary objective of this study was to examine the effects of two “feline friendly” recommendations, minimized waiting room experience, and FFP, on blood pressure measurements. We hypothesized that bypassing the waiting room and the use of FFP would be associated with lower blood pressure values. We also hypothesized that cats would acclimate to the experience over time, resulting in lower blood pressure values over subsequent clinic visits.

## Materials and Methods

This research was reviewed and approved by the Institutional Animal Care and Use Committee at Iowa State University (Protocol ID#: 4-16-8249-F).

### Study Design

A 2 × 2 factorial design was used to compare effects of patient waiting location and exposure to a spray containing a synthetic analog of the F3 fraction of FFP (Feliway™, Ceva Animal Health, Lenexa, KS) on blood pressure in cats. To control for potential effects of individual cats, including their temperament, all cats received all treatments. Additionally, treatment order for each cat was randomized. Randomization procedures were completed using a random number generator. To avoid subjective bias, personnel collecting blood pressure data were blind to treatment allocations for all cats; for practical reasons, cat owners, and personnel collecting vocalization data could not be blinded to treatment. All cats received treatments singly, and hence cat was the experimental unit.

### Experimental Procedures

This study was conducted at the Lloyd Veterinary Medical Center, Iowa State University (ISU) College of Veterinary Medicine (CVM) during normal business hours from 1,300 to 1,700 h, 2016 and 2017.

Treatments were administered in the following combinations, with cats arriving in and remaining in carriers provided by their owners. Waiting room (WR+) treatment consisted of a 10-min dwell time in the designated hospital waiting room, after which the client and patient were escorted to the examination room. The carrier was placed on a chair with the carrier door facing outwards to the waiting area. Bypassed waiting room (WR–) treatment consisted of a 10-min dwell time in the examination room. In the examination room, the carrier was placed on a chair with the door facing outwards toward interior of the room. A large beach towel, provided by the researchers and sprayed with FFP, was placed over the carrier during the dwell time (FFP+). Control (FFP–) treatment consisted of an identical beach towel without FFP spray in 2016, and no beach towel or FFP spray in 2017. No instructions were provided to owners regarding provision of FFP, bedding, or towels inside the carriers. During the 10-min dwell period, the owner filled out a brief patient medical history. If an owner arrived much earlier than scheduled, a neutral holding place (a closed, quiet office with no human or animal traffic) was provided for them until the cat could be enrolled in its assigned treatment. Following the 10-min dwell period, the carrier was placed on the examination table, and an additional 10 min (the “acclimatization period”) was provided to allow the cat to acclimate before the exam. This was done because a 5–10 min acclimatization period to the measurement room is recommended prior to blood pressure evaluation (6, 7). One researcher recorded the number of vocalizations performed by the cat during the acclimation period.

The clinical exam period began when a second researcher responsible for blood pressure measurements entered the examination room, and removed the cat from the carrier. A Parks Doppler Flow Detector Model Number 811-B with cuff sizes 2–4 cm was used to measure blood pressure. To eliminate audible static, headphones were worn by the researcher whenever possible. Due to equipment malfunction, headphones were periodically unavailable. Headphones were used for blood pressure measurement in 78 out of 88 visits (89%) in 2016 and in 46 out of 68 visits (68%) in 2017. The tail base or rear leg was measured to determine cuff size (40% of appendage circumference) (21). The tail base was used as the primary site, but the rear leg was used when tails were too short or tail manipulation was not tolerated by the cat. The blood pressure site chosen for each cat was consistently used across all four visits. Hair was clipped on the first visit to facilitate identification of the pulse prior to placement of the Doppler probe, and was re-clipped as needed during subsequent visits. Six readings were obtained on each visit, according to the method previously described (21). The first reading was excluded, and mean blood pressure was calculated using the remaining readings. Mean blood pressure was categorized according to clinical relevance, where normotensive (NT) was identified as <150 mmHg, borderline hypertensive/hypertensive (BHT/HT) as 150–179 mmHg, and severely hypertensive (SHT) as ≥180 mmHg (6, 7). Time required to complete the blood pressure measurements was collected from video recordings during 2016 and was manually recorded in 2017.

After the blood pressure measurements were recorded, a brief physical exam was performed, including heart rate, respiratory rate, body condition score, and body weight. Heart rate was determined by auscultation with a stethoscope with the number of beats counted in 15 s and multiplied by four. Respiratory rate was determined by observing thoracic excursions and counting the number of complete breaths (inspiration and expiration) in 15 s and multiplying by four. Body condition score was determined using a nine-point scale (22). Blood pressure and physical exam were performed using minimal restraint when possible (6); scruffing was discouraged (23). Cuff sizes and position, restraint techniques, carrier type, and body position for blood pressure measurements were noted and kept consistent for subsequent appointments (21). All technicians handling cats were trained to use low stress handling techniques as recommended by the *Low Stress Handling for Dogs and Cats*® (Cattledog Publishing, Davis, CA), and training was provided by a veterinarian (JC), Certified Silver level in this program (2015) and certified in the Cat-Friendly Practice® AAFP program (2015).

### Experimental Animals

Cats were recruited from faculty, staff, and students of ISU in summer 2016 and 2017, with the following inclusion criteria based on owner self-reporting: at least 1 year of age, no chronic illnesses, not on medications with potential to affect blood pressure, and no history of aggressive behavior during routine veterinary visits. All owners provided written, informed consent at the first appointment.

*A priori* sample size calculations determined that 31 cats would provide 80% statistical power to detect a 15 mmHg difference in blood pressure. Published feline blood pressure data (24) were used as estimates of clinical variability.

### Statistical Analysis

Data distributional properties were examined using Proc Univariate. Blood pressure, time to collect blood pressure, heart rate, and respiration rate data were normal, whereas vocalization data were skewed. Vocalizations were collected and analyzed as count data. All data (blood pressure, blood pressure categories, time to collect blood pressure, heart rate, respiration rate, and vocalizations) were analyzed using SAS® software with generalized linear mixed models (GLIMMIX) to compare differences in outcomes based on WR and FFP treatments. A Gaussian distribution was used for blood pressure, heart rate, and respiration rate. A binary distribution was used to analyze occurrence of vocalization (Yes/No), and a Poisson distribution was used for frequencies of vocalizations. Time to collect blood pressure was analyzed separately for each year as the methodology for collecting these data differed between the 2 years.

The statistical models included the fixed effects of WR and FFP interactions, visit, year, and sex, with the exception of time to collect blood pressure, which included the fixed effects WR and FFP interactions, visit, and sex. Cat age and weight were included as covariates, but were later removed from the models due to lack of significance. Cat ID was included in the model as subject in the random statement.

The I-Link option was used to transform LSMeans and standard errors back to original units for respective measurements and subsequent reporting. *P* ≤ 0.05 was considered significant.

## Results

Forty-eight cats were enrolled in the study (26 in 2016, 22 cats in 2017). Nine cats were ultimately excluded from the study (four in 2016, five in 2017). One cat was removed from the study at the owner's request after the first visit when hypertension was discovered and anti-hypertensive therapy was implemented. Another cat was removed due to extreme resistance to handling. The remaining cats were removed because they did not complete all four visits. Hence, 39 cats remained in the dataset for the final analyses (median age: 4 y; age range: 1–15 y; median weight: 5.9 kg; weight range: 3–8.4 kg; sex: 17 neutered males and 22 spayed females; breed: 34 domestic shorthairs, 3 domestic longhairs, 1 Siamese mix, and 1 Persian).

Cats that arrived at the clinic prior to their assigned appointment (*n* = 3 visits in 2016) were kept in a neutral holding area. Minimal restraint included toweling during 18 visits in 2016 (21% of visits), whereas toweling was used only three times in 2017 (4% of visits). Scruffing was used for one cat during one visit in 2016 (1% of visits), and on 14 visits (21% of visits) in 2017. Full body restraint was not used on any cats. For one cat, blood pressure data could not be collected on visit 1 due to equipment malfunction, but all other parameters were measured.

**Figure 1:**
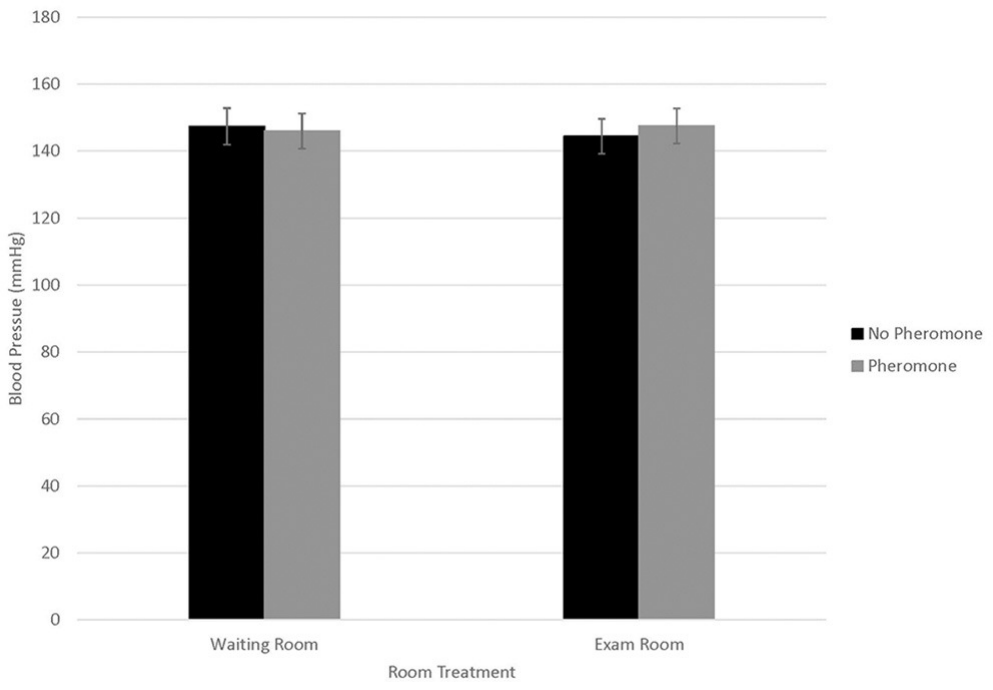
LSMean (±SE) feline blood pressure (mmHg) as assessed using Doppler^1^ at a veterinary clinic. Over four visits, 39 cats^2^ were randomly assigned to one of four possible treatment combinations, (WR+/FFP+, WR+/FFP–, WR–/FFP+, WR–/FFP–)^3^ which were experienced for 10-min prior to the acclimation period and blood pressure measurements. ^1^Parks Doppler Flow Detector Model Number 811-B with cuff sizes 2–4 cm was used to measure blood pressure. The tail base the primary site, but the rear leg was used when tails were too short or tail manipulation was not tolerated by the cat. The site chosen for each cat was used across all four visits. Hair was clipped on the first visit to facilitate identification of the pulse prior to placement of the Doppler probe, and was re-clipped as needed during subsequent visits. ^2^All enrolled cats were mature, healthy, and either spayed or neutered. ^3^WR+: owners and their cats stayed in the waiting room lobby at veterinary clinic for 10 min prior to being taken into the exam room for blood pressure measurements; WR–: owners and their cats were immediately taken into the exam room where they waited for 10 min prior to blood pressure measurements; FFP+: a clean towel was sprayed with feline facial pheromone and placed in their carriers during the 10 min acclimation period; FFP–: no feline facial pheromone was provided.

**Figure 2:**
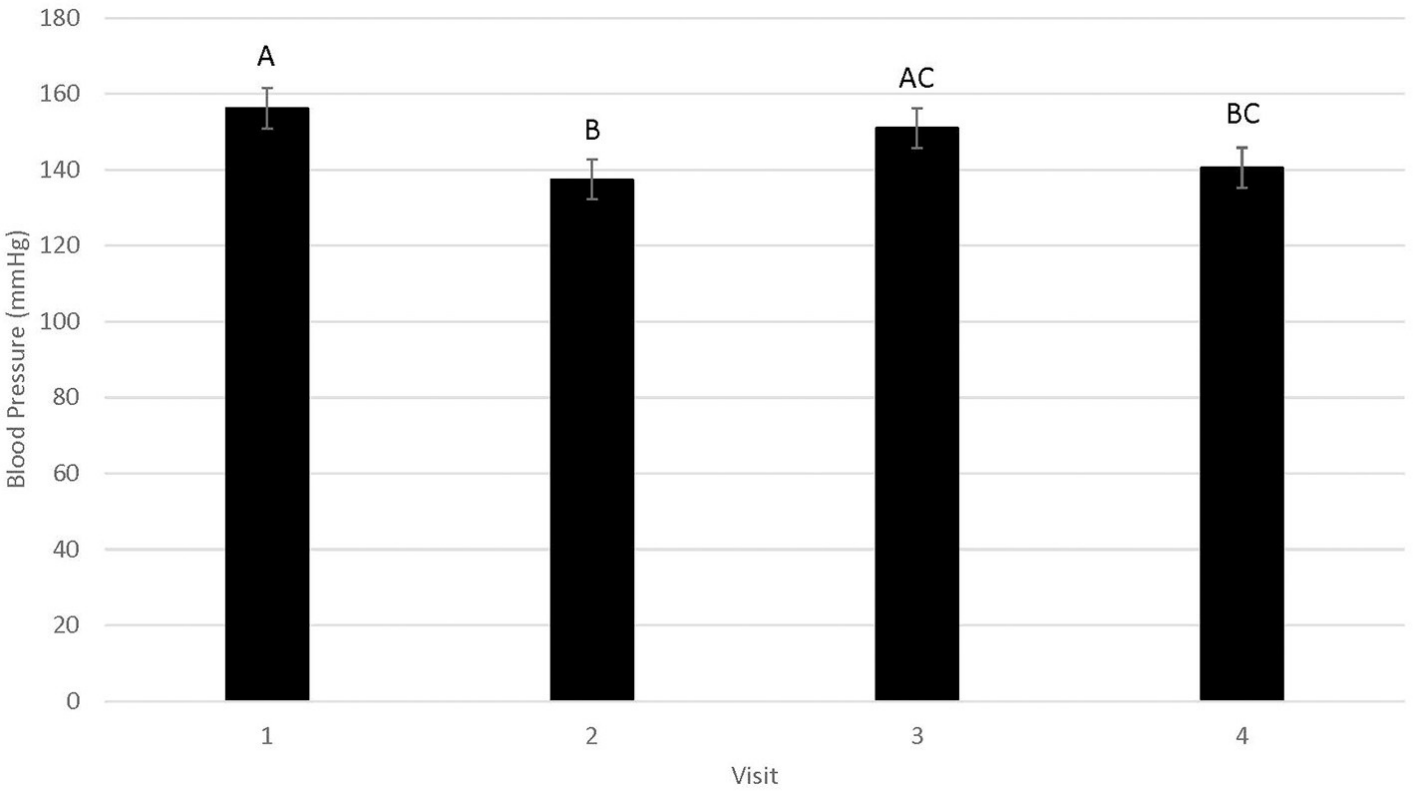
LSMean (±SE) feline blood pressure (mmHg) as assessed using Doppler^1^ during four visits to a veterinary clinic. Columns letters denote statistical differences between visits (*P* < 0.05). ^1^Parks Doppler Flow Detector Model Number 811-B with cuff sizes 2–4 cm was used to measure blood pressure. The tail base the primary site, but the rear leg was used when tails were too short or tail manipulation was not tolerated by the cat. The site chosen for each cat was used across all four visits. Hair was clipped on the first visit to facilitate identification of the pulse prior to placement of the Doppler probe, and was re-clipped as needed during subsequent visits. ^2^All enrolled cats were mature, healthy, and either spayed or neutered.

Blood pressure data were statistically analyzed using all six readings, as well as using only readings 2–6 as per clinical recommendations; since no statistical differences were identified for these approaches, results from readings 2–6 are reported. Differences in blood pressure were not associated with WR by FFP interactions (Figure 1; *n* = 0.95). Similarly, blood pressure did not differ by year (146.3 ± 5.5; *n* = 0.15). Blood pressure differed by visit (Figure 2; *P* < 0.01), but not in a consistent manner. Higher blood pressure was associated with visit 1 relative to visit 2 (*P* < 0.01) and visit 4 (*n* = 0.01), whereas higher blood pressure was associated with visit 3 relative to visit 2 (*n* = 0.02). Males displayed higher blood pressure than females (156.6 ± 5.77 mmHg and 136.0 ±5.20 mmHg, respectively; *n* = 0.01).

Out of a total of 155 visits during which blood pressure was successfully measured, cats were categorized as NT during 97 visits, BHT/HT during 39 visits and SHT during 19 visits. Of those cats receiving a classification of BHT/HT during the study, 14 cats received this classification once, nine cats on two visits, one cat on three visits, and one cat on four visits. Of the cats receiving a classification of SHT during the study, six cats received this classification on one visit, five cats on two visits, and one cat on three visits. There were no observed effects of WR by FFP interactions, visit or year on blood pressure categories (*P* > 0.09). Blood pressure categories differed between males and females, with males having a greater probability of being placed in a higher blood pressure category than females (1.7 ± 0.16 and 1.3 ± 0.13, respectively; *n* = 0.01).

The time (in minutes) needed to obtain the blood pressure measurements was not associated with WR by FFP interaction (2016 = 5.4 ± 0.6, 2017 = 2.4 ± 0.4). In 2016, visit 1 (8.4 ± 0.7) was significantly longer than subsequent visits (5.3 ± 0.6; *P* < 0.01) and there was a trend for males to take longer than females (6.7 ± 0.5 and 5.5 ± 0.4, respectively; *n* = 0.06). In 2017, no differences were observed for visit or sex (2.4 ± 0.4 and 2.4 ± 0.3, respectively; *P* > 0.26).

Heart rate was not associated with WR by FFP interaction, visit, year, or sex (187.2 ± 4.70, 187.2 ± 4.70, 187.2 ± 5.46, 187.2 ± 5.46 bpm, respectively; *P* > 0.07). Similarly, respiratory rate was not associated with WR by FFP interaction or visit (59.80 ± 2.86 for both effects; *P* > 0.16). Respiration rate differed by sex, with males having a lower respiration rate (55.3 ± 3.33) than females (64.3 ± 2.98, *P* < 0.05). Respiration also differed by year, with 2016 having higher respiration rates (72.3 ± 2.98) than 2017 (47.3 ± 3.33, *P* < 0.01).

**Figure 3:**
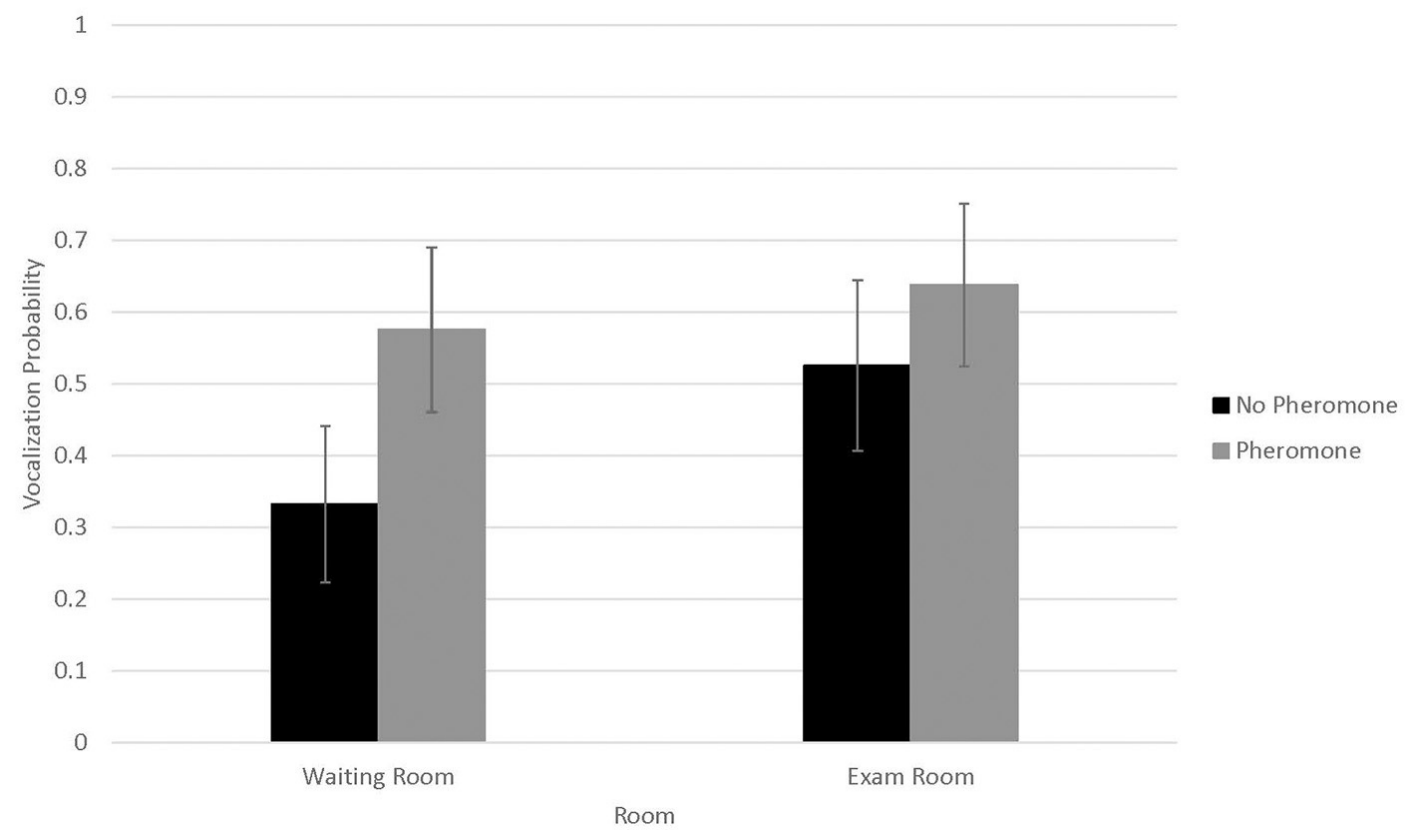
LSMean (±SE) feline vocalization probabilities during a 10-min acclimation period in a veterinary clinical exam room. Over four visits, 39 cats^1^ were randomly assigned to one of four possible treatment combinations (WR+/FFP+, WR+/FFP–, WR–/FFP+, WR–/FFP–),^2^ which were experienced for 10-min prior to the acclimation period and blood pressure measurements. ^1^All enrolled cats were mature, healthy, and either spayed or neutered. ^2^WR+: owners and their cats stayed in the waiting room lobby at veterinary clinic for 10 min prior to being taken into the exam room for blood pressure measurements; WR–: owners and their cats were immediately taken into the exam room where they waited for 10 min prior to blood pressure measurements; FFP+: a clean towel was sprayed with feline facial pheromone and placed in their carriers during the 10 min acclimation period; FFP–: no feline facial pheromone was provided.

**Figure 4:**
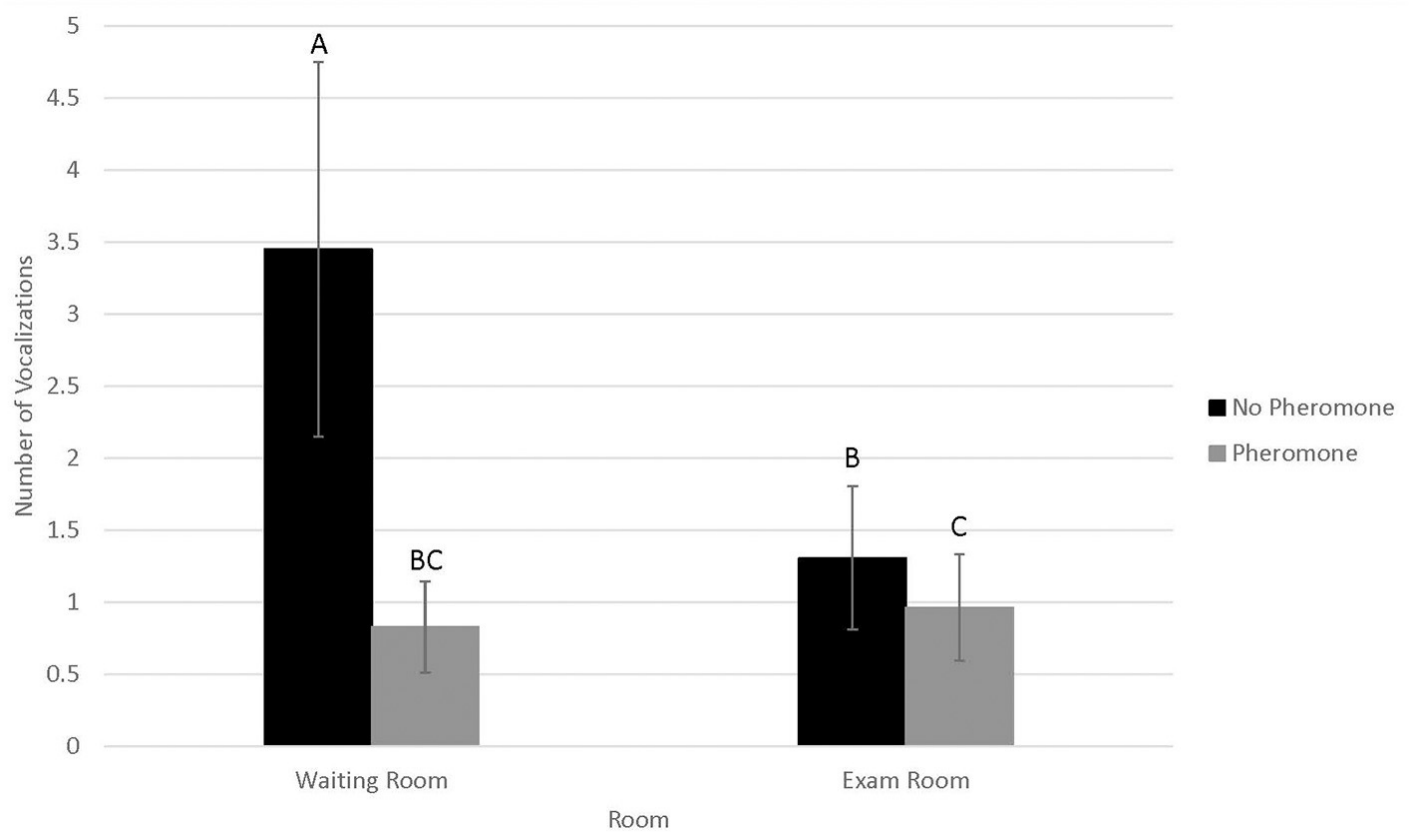
LSMean (±SE) number of feline vocalizations during a 10-min acclimation period in a veterinary clinical exam room. Over four visits, 39 cats^1^ were randomly assigned to one of four possible treatment combinations (WR+/FFP+, WR+/FFP–, WR–/FFP+, WR–/FFP–),^2^ which were experienced for 10-min prior to the acclimation period and blood pressure measurements. Columns letters denote statistical differences between visits (*P* < 0.05). ^1^All enrolled cats were mature, healthy, and either spayed or neutered. ^2^WR+: owners and their cats stayed in the waiting room lobby at veterinary clinic for 10 min prior to being taken into the exam room for blood pressure measurements; WR–: owners and their cats were immediately taken into the exam room where they waited for 10 min prior to blood pressure measurements; FFP+: a clean towel was sprayed with feline facial pheromone and placed in their carriers during the 10 min acclimation period; FFP–: no feline facial pheromone was provided.

Probability of vocalizing (Figure 3) was not associated with WR by FFP interactions (*N* = 0.14) or visit (*n* = 0.72). However, probability of vocalizing was higher in 2016 (0.73 ± 0.09) than 2017 (0.30 ± 0.10, *P* < 0.01). Conversely, number of vocalizations (Figure 4) was significantly associated with WR by FFP interactions (*P* < 0.01), with fewer vocalizations associated with WR– and FFP+ treatments (*P* < 0.01). Number of vocalizations was associated with visit (*n* = 0.02); fewer vocalizations were observed on visits 1 and 2 than visit 3 (1.2 ± 0.5, 1.3 ± 0.5, and 1.6 ± 0.6, respectively; *P* < 0.04). Number of vocalizations was not associated with sex (*n* = 0.20), but more vocalizations were observed in 2017 (3.09 ± 1.7) than in 2016 (0.6 ± 0.3; *n* = 0.03).

## Discussion

Blood pressure measurement in the cat is a process that is fraught with difficulty. In the stress of a clinical setting, blood pressure can increase above what may be considered normal in a home environment (10, 12), making clinical interpretation of blood pressure challenging. In this study, we evaluated the effect of two purported low-stress interventions, bypassing the WR and FFP spray, and found no statistically significant effects on blood pressure in cats.

Blood pressure measurements obtained in this study were similar to or slightly higher than those reported using Doppler in healthy cats (10, 13, 24, 25). Because these studies were all performed using forelimb as the primary site of blood pressure measurement, our use of the tail as the primary site may account for these differences. It has been shown that measuring blood pressure using the tail results in higher values than when the forelimb is used (26–28). We also found male cats had a higher blood pressure than females, which is consistent with other studies (13).

For the purposes of our study, we classified blood pressure according to the following categories: <150: NT; 150–179: borderline hypertensive/hypertensive (BHT/HT); ≥180: SHT. This is roughly based on ISFM guidelines and historic American College of Veterinary Internal Medicine (ACVIM) hypertension guidelines, although the updated ACVIM guidelines now classify 140–159 mmHg as pre-hypertensive (6, 7, 29). Average blood pressure per visit was ≥150 mmHg in 37% of visits, which would warrant re-evaluation for true hypertension. In 12% of visits the blood pressure was ≥180 mmHg, which would have suggested that treatment was indicated. Despite the fact that abnormally high blood pressure readings were recorded frequently during our study, only five out of the 39 cats evaluated were classified as either BHT/HT or SHT consistently across the four visits. This supports the recommendation that, in the absence of clear evidence of target organ damage, repeated blood pressure measurements should be performed at subsequent visits prior to making the decision to treat hypertension (6, 7).

We anticipated that bypassing the waiting room and the use of FFP would reduce stress in feline patients, translating to lower and more reliable blood pressure measurements. There was some evidence to support this assumption of reduced stress, in terms of vocalizations. Struggling and vocalization in cats have been associated with sympathoadrenal stimulation, and hence expected to result in an increase in blood pressure (30).

Vocalizations occurred in one-third to two-thirds of all visits, and the number of vocalizations performed were roughly three times greater when cats did not bypass the WR and were not provided with FFP. Since our experimental design and statistical analysis controlled for confounding effects associated with individual cat responses to stress, these differences were likely driven by the treatment interventions. However, it is important to note that vocalizations were collected during the waiting period, and were not recorded during the physical exam or blood pressure measurement procedures. Treatment differences were not observed for physiological responses to stress, including blood pressure, during the examination itself. Hence, if indeed bypassing the waiting room and use of FFP reduced stress in the feline patients, the effects if present may be short lived and did not translate to improved blood pressure as we had hoped.

Although not significant, occurrence of vocalization numerically showed similar but opposite associations with treatment combination; vocalizations were less likely to occur when cats did not bypass the WR and were not provided with FFP. It is possible that distress may have suppressed vocalization occurrence entirely, whereas vocalization frequency was exacerbated for individuals that displayed this behavior. Relationships between vocalizations and distress in cats are variable in the scientific literature. Some researchers reported vocalization score was a reliable indicator of stress when cats were restrained in a handling carrier, showing intra-cat response consistency over repeated exposure to this acute stressor (31). In contrast, others report fearful cats to perform fewer vocalizations in an open -field test than non-fearful or mildly fearful cats (32). Since we did not discriminate between types of vocalizations, a more refined ethogram or spectrogram capable of teasing out high frequency calls characteristic of fear and distress from other types of vocalizations would have been useful (33). Similarly, cognitive tests such as conditioned avoidance may have aided interpretation of the discrepancy of these results in terms of affective responses (8). However, these refinements were beyond the scope of the current study.

The WR is identified as a source of stress in cats during visits to the veterinary clinic, which may alter physiologic parameters such as blood pressure, and has been associated with increased stress behaviors as perceived by cat owners, particularly in response to the presence of dogs and other animals (9). While no studies specifically report effects of bypassing the WR in cats, dogs displayed higher cortisol and heart rate in a veterinary WR than in a yard environment (15). It is reasonable to suspect that cats may display a similar or even a more exaggerated stress response than dogs, since most cats do not routinely venture outside of their home environment and are not as often exposed to unfamiliar people and animals. Cats display higher respiratory rates and heart rates in the veterinary environment than in the home (10, 11). However, effects of evaluating blood pressure outside of the veterinary hospital environment are less clear, with Conti et al. (11) finding no difference between the two environments and Quimby et al. (10) finding a small increase in blood pressure in the veterinary hospital environment that was deemed clinically insignificant. Interestingly, cats display more vocalization and struggling in response to handling during medical evaluations in a home environment vs. in a veterinary environment (10, 11).

It is possible that WR had no effect on blood pressure in this study because the experiences associated with the waiting and exam rooms may not have sufficiently differed. This study took place at a large veterinary referral hospital that has a partitioned WR intended to separate species. Efforts were made during our study to have cats wait in an environment that would be typical of a small waiting area in a veterinary clinic that includes other cats, dogs, and other species in close proximity. Because study cases were seen in the afternoon when hospital caseload was often lighter, it is possible that the WR environment was unnaturally quiet. This information was not recorded, and would have aided interpretation of our findings.

It is also possible that WR did not affect blood pressure in the cats in this study because they were allowed an acclimation period once they entered the exam room. In a previous study evaluating 24-h ambulatory blood pressure in cats, transitions into the waiting room and into the exam room were both associated with a transient increase in blood pressure (12). Novelty associated with transition to a new environment may result in increased blood pressure rather than the choice of environment; however, we saw no compelling evidence of habituation over the four visits. Hence, including an acclimation period in the exam room may be more important than avoiding the WR when it comes to effects on blood pressure specifically.

Studies evaluating whether FFP can help reduce behavioral indictors of stress in a veterinary setting have shown mixed results. Griffith et al. showed a decrease in anxiety-related behavior and increased food intake in a hospital setting in association with FFP (19). Some studies have shown an increase in calm behaviors associated with FFP, but no difference in ease of handling (34, 35). However, Conti et al. found no significant difference in behavior scores when FFP was used with cats in a clinical setting, and found FFP spray in home and hospital environments did not affect blood pressure (11). Interestingly, participants in a blinded parallel study reported improved cat–dog interactions and greater cat relaxation in multi-pet households when a pheromone diffuser was used, and these effects were observed in association with FFP and dog appeasement pheromone (Adaptil™) products (36).

Other factors associated with the visit, independent of WR and FFP may have contributed to the cats in our study being maximally stressed and unaffected by these interventions. Cats have been shown to experience stress during all stages of the veterinary visit, including transportation to the clinic and the time leading up to departure from the home environment (9, 12). While all cats were handled with minimal restraint for blood pressure measurement in accordance with published guidelines (6, 7), the act of obtaining the blood pressure measurements likely contributed to stress in our cats. Two studies evaluating blood pressure in cats in a home and hospital setting did not find a clinically significant difference in blood pressure between the two environments, leading to the suspicion that restraint and struggling associated with obtaining blood pressure measurements contributes as much to patient stress as the environment in which it is taken (10, 11). It is also possible that our cat population was unusually unaffected by typical stressors associated with the veterinary visit. The cats enrolled in the study were owned primarily by students and staff, many of which had participated in other studies.

There was a significant difference in vocalizations by year with fewer vocalizations across visits and treatments in 2016 than in 2017. This is particularly noteworthy since time to collect blood pressure measurements was more than twice as long in 2016. Conversely, the likelihood that cats would vocalize at least once during the visit was higher in 2016. Several factors that may have contributed to these discrepancies, including the possibility that the cohort of cats in 2017 were inherently more vocal. In response to novel stimuli in test situations, feral cats display a higher vocalization rate than socialized house cats, in conjunction with extreme aggressive and defensive behaviors consistent with fear and distress affective states (37). These vocalizations were also longer in duration, with different spectrogram features in that study. Unfortunately, we were unable to capture these details in our study, which would aid interpretation of the underlying affective states. An additional towel was not placed over the carrier in the FFP- treatment in 2017, which may have resulted in differences in stress during the waiting period. Furthermore, personnel differed between the two summers over which the study was conducted. While handlers were instructed to use minimal restraint and toweling techniques if extra restraint was necessary, scruffing was used with greater frequency in 2017. It is possible that cats in 2017 were more fractious, resulting in greater use of the discouraged restraint techniques of scruffing; nonetheless, it is likely that the handling method in 2017 resulted in greater stress experienced (8, 23). When possible, headphones were used during blood pressure measurements to prevent the cats from hearing and possibly becoming startled by the sounds made by the Doppler. Due to equipment failure, headphones could not be used consistently; headphones were used more frequently in 2016 than in 2017. While it has previously been suggested that the use of headphones for Doppler does not affect blood pressure in the cat, it is still possible, and the effect of Doppler noise on stress responses such as behavior has not been evaluated (38). It is possible that the noise produced by the Doppler did have an effect on the stress experienced by cats in our study, resulting in a higher vocalization rate in 2017 when headphones were used less frequently.

Mean blood pressure did differ across visits, with mean blood pressure in visit 1 significantly higher than mean blood pressure in visits 2 and 4, but not visit 3. While a higher blood pressure in visit 1 could suggest acclimation to multiple veterinary visits over a short period of time, the fact that there was not a progressive decline in mean blood pressure and that blood pressure in visit 3 did not differ from visit 1 suggests that cats did not acclimate. These results are consistent with a previous study, in which cats did not have a lower blood pressure with multiple simulated veterinary visits (12). Additionally, visit did not affect occurrence or frequency of vocalizations in our cats, further supporting the lack of acclimation to repeated veterinary visits. It is suspected that cats had a higher blood pressure in visits 1 and 3 because fur was clipped to facilitate identification of an audible pulse with the Doppler probe. The fur was clipped on visit 1 in all cats and reclipped as needed throughout the study with regrowth of hair; cats typically required clipping again on visit 3. Clipping may have been a source of stress for the cats and could have contributed to higher blood pressure on this visit.

In conclusion, results of this study suggest that the use of FFP and bypassing the WR do not result in lower blood pressure measurements in cats. Although we found a large number of clinically healthy cats presenting with blood pressure measurements high enough to warrant treatment, these values were infrequently repeatable across multiple visits, suggesting blood pressure measurement should be interpreted with caution in the absence of risk factors for hypertension. While blood pressure was not affected, we cannot decisively comment on the stress experienced by cats in this study, and the degree to which these interventions mitigated stress. More detailed analysis of stress, including behavioral responses, physiologic responses, and cognitive effects could be evaluated in future studies to inform best practices for mitigating fear and distress in the clinical environment.

## Data Availability Statement

The original contributions presented in the study are included in the article/Supplementary Material, further inquiries can be directed to the corresponding author/s.

## Ethics Statement

The animal study was reviewed and approved by Institutional Animal Care and Use Committee at Iowa State University. Written informed consent was obtained from the owners for the participation of their animals in this study.

## Author Contributions

The idea for the study and the experimental design were developed by JC, SM, and LV. Data was collected by MR, JC, RP, and LV. Data was analyzed by SM and RP. All authors contributed to writing and final approval of the manuscript.

## Conflict of Interest

The authors declare that the research was conducted in the absence of any commercial or financial relationships that could be construed as a potential conflict of interest.

## References

[1] Kobayashi DL Peterson ME Graves TK Lesser M Nichols CE. Hypertension in cats with chronic renal failure and hyperthyroidism. J Vet Intern Med. (1990) 4:58–62. 10.1111/j.1939-1676.1990.tb03104.x 2342023

[2] Bodey AR Sansom J. Epidemiological study of blood pressure in domestic cats. J Small Anim Pract. (1998) 39:567–73. 10.1111/j.1748-5827.1998.tb03710.x 9888110

[3] Elliot J Barber PJ Syme HM Rawlings JM Markwell PJ. Feline hypertension: clinical findings and response to antihypertensive treatment in 30 cases. J Small Anim Pract. (2001) 42:122–129. 10.1111/j.1748-5827.2001.tb02008.x 11303854

[4] Jepson RE. Feline systemic hypertension. Classification and pathogenesis. J Feline Med Surg. (2011) 13:25–34. 10.1016/j.jfms.2010.11.007 21215946 PMC10845409

[5] Stepien RL. Feline systemic hypertension. Diagnosis and management. J Feline Med Surg. (2011) 13:35–43. 10.1016/j.jfms.2010.11.008 21215947 PMC10845410

[6] Taylor SS Sparkes AH Briscoe K Carter J Cervantes Sala S Jepson RE. ISFM consensus guidelines on the diagnosis and management of hypertension in cats. J Fel Med Surg. (2017) 19:288–303. 10.1177/1098612X17693500 28245741 PMC11119534

[7] Acierno MJ Brown S Coleman AE Jepson RE Papich M Stepien RL. ACVIM consensus statement: guidelines for the identification, evaluation, and management of systemic hypertension in dogs and cats. J Vet Intern Med. (2018) 32:1803–22. 10.1111/jvim.15331 30353952 PMC6271319

[8] Moody CM Picketts VA Mason GJ Dewey CE Niel L. Can you handle it? Validating negative responses to restraint in cats. Appl Anim Behav Sci. (2018) 204:94–100. 10.1016/j.applanim.2018.04.012

[9] Mariti C Bowen JE Campa S Grebe G Sighieri C Gazzano A. Guardians' perceptions of cats' welfare and behavior regarding visiting veterinary clinics. J Appl Anim Welf Sci. (2016) 19:375–384. 10.1080/10888705.2016.1173548 27116303

[10] Quimby JM Smith ML Lunn KF. Evaluation of the effects of hospital visit stress on physiologic parameters in the cat. J Fel Med Surg. (2011) 13:733–7. 10.1016/j.jfms.2011.07.003 21839664 PMC10832771

[11] Conti LMC Champion T Guberman UC Mathias CHT Fernandes SL Silva EGM. Evaluation of environment and a feline facial pheromone analogue on physiologic and behavioral measures in cats. J Fel Med Surg. (2015) 19:165–70. 10.1177/1098612X15621107 26662036 PMC10816560

[12] Belew AM Bartlett T Brown SA. Evaluation of the white-coat effect in cats. J Vet Intern Med. (1999) 13:134–142. 10.1111/j.1939-1676.1999.tb01141.x 10225603

[13] Payne JR Brodbelt DC Luis Fuentes V. Blood pressure measurements in 780 apparently healthy cats. J Vet Intern Med. (2017) 31:15–21. 10.1111/jvim.14625 27906477 PMC5259628

[14] Rodan I Sundahl E Carney H Gagnon A Heath S Landsberg G. AAFP and ISFM feline-friendly handling guidelines. J Fel Med Surg. (2011) 13:364–75. 10.1016/j.jfms.2011.03.012 21515223 PMC11107994

[15] Perego R Proverbio D Spada E. Increases in heart rate and serum cortisol concentrations in healthy dogs are positively correlated with an indoor waiting-room environment. Vet Clin Pathol. (2013) 43:67–71. 10.1111/vcp.12118 24446821

[16] Mariti C Raspanti E Zilocchi M Carlone B Cazzano A. The assessment of dog welfare in the waiting room of a veterinary clinic. Anim Welfare. (2015) 24:299–305. 10.7120/09627286.24.3.299

[17] Hetts S Heinke ML Estep DQ. Behavior wellness concepts for general veterinary practice. J Am Vet Med Assoc. (2004) 225(4):506–13. 10.2460/javma.2004.225.506 15344354

[18] Carney HC Little S Brownlee-Tomasso D Harvey AM Mattox E Robertson S. AAFP and ISFM feline-friendly nursing care guidelines. J Fel Med Surg. (2012) 14:337–49. 10.1177/1098612X12445002 22511476 PMC11132257

[19] Griffith CA Steigerwald ES Buffington CA. Effects of a synthetic facial pheromone on behavior of cats. J Am Vet Med Assoc. (2000) 217(8):1154–6. 10.2460/javma.2000.217.1154 11043684

[20] Patel G Heath S Coyne K German AC. Pilot study to investigate whether a feline pheromone analogue reduces anxiety-related behavior during clinical examination of cats in a rescue shelter. J Vet Behav. (2010) 5:33. 10.1016/j.jveb.2009.10.022

[21] Henik RA Dolson MK Wenholz LJ. How to obtain a blood pressure measurement. Clin Tech Small Anim Pract. (2005) 20:144–50. 10.1053/j.ctsap.2005.05.005 16180396

[22] LaFlamme DP. Development and validation of a body condition score system for cats: a clinical tool. Feline Pract. (1997) 25(5-6):13–18.

[23] Moody CM Mason GJ Dewey CE Niel L. Getting a grip: cats respond negatively to scruffing and clips. Vet Rec. (2020) 186:385. 10.1136/vr.105261 31586939

[24] Sparkes AH Caney SM King MC Gruffydd-Jones TJ. Inter- and intraindividual variation in Doppler ultrasonic indirect blood pressure measurements in healthy cats. J Vet Intern Med. (1999) 13:314–318. 10.1111/j.1939-1676.1999.tb02187.x 10449221

[25] Lin C Yan C Lien Y Huang H. Systolic blood pressure of clinically normal and conscious cats determined by an indirect Doppler method in a clinical setting. J Vet Med Sci. (2006) 68:827–32. 10.1292/jvms.68.827 16953083

[26] Zeugswetter FK Tichy A Weber K. Radial vs. coccygeal artery Doppler blood pressure measurement in conscious cats. J Fel Med Surg. (2017) 20:968–72. 10.1177/1098612X17740795 29132245 PMC11129233

[27] Whittemore JC Nystrom MR Mawby DI. Effects of various factors on Doppler ultrasonographic measurements of radial and coccygeal arterial blood pressure in privately owned, conscious cats. J Am Vet Med Assoc. (2017) 250:763–9. 10.2460/javma.250.7.763 28306487

[28] Cannon MJ Brett J. Comparison of how well conscious cats tolerate blood pressure measurement from the radial and coccygeal arteries. J Fel Med Surg. (2012) 14:906–9. 10.1177/1098612X12455023 22832888 PMC11108010

[29] Brown S Atkins C Bagley R Carr A Cowgill L Davidson M. Guidelines for the identification, evaluation, and management of systemic hypertension in dogs and cats. J Vet Intern Med. (2012) 21:542–58. 10.1892/0891-6640(2007)21[542:gftiea]2.0.co;2 17552466

[30] Rand JS Kinnaird E Baglioni A Blackshaw J Priest J. Acute stress hyperglycemia in cats is associated with struggling and increased concentrations of lactate and norepinephrine. J Vet Intern Med. (2002) 16:123–32. 10.1892/0891-6640(2002)016<0123:ashici>2.3.co;2 11899027

[31] Urrutia A Martinez-Byer S Szenczi P Hudson R Banszegi O. Stable individual differences in vocalisation and motor activity during acute stress in the domestic cat. Behav Process. (2019) 165:58–65. 10.1016/j.beproc.2019.05.022 31132445

[32] de Rivera C Ley J Milgram B Landsberg G. Development of a laboratory model to assess fear and anxiety in cats. J Feline Med Surg. (2017) 19:586–93. 10.1177/1098612X16643121 27090290 PMC11128803

[33] Tavernier C Ahmed S Houpt KA Yeon SC. Feline vocal communication. J Vet Sci. (2020) 21:*e*18. 10.4142/jvs.2020.21.e18 PMC7000907 32017479

[34] Kronen PW Ludders JW Erb HN Moon PF Gleed RD Koski S. A synthetic fraction of feline facial pheromones calms but does not reduce struggling in cats before venous catheterization. Vet Anaesths Analg. (2006) 33:258–65. 10.1111/j.1467-2995.2005.00265.x 16764591

[35] Pereira JS Fragoso S Beck A Lavigne S Veregao AS Pereira G. Improving the feline veterinary consultation: the usefulness of Feliway spray in reducing cats' stress. J Fel Med Surg. (2015) 18:959–64. 10.1177/1098612X15599420 26282847 PMC11112237

[36] Prior MR Mills DS. Cats vs. dogs: the efficacy of Feliway FriendsTM and AdaptilTM products in multispecies homes. Front Vet Sci. (2020) 7:399. 10.3389/fvets.2020.00399 32754622 PMC7366870

[37] Yeon SC Kim YK Park SJ Lee SS Lee SY Suh EH. Differences between vocalization evokes by social stimuli in feral cats and house cats. Behav Process. (2011) 87:183–9. 10.1016/j.beproc.2011.03.003 21443933

[38] Williams TL Elliott J Syme HM. Measurement of systolic blood pressure (SBP) in cats by the indirect Doppler technique is not altered by the use of headphones. In: ECVIM Congress (2010).

