# Supplementary material for: Effects of Waiting Room and Feline Facial Pheromone Experience on Blood Pressure in Cats
Source: Front Vet Sci. 2021 Mar 5;8:640751. doi: 10.3389/fvets.2021.640751 (PMC7973014; doi:10.3389/fvets.2021.640751)
Supplement: Supplementary file 1 [file Data_Sheet_1.PDF]

Appendix 1: Blood pressure and vocalization data for all cats by visit. WR=waiting room; FFP=feline facial pheromone; BP=blood pressure.

Cat ID	Visit	WR	FFP	BP mean	Vocalizations
**4**	1	Y	Y	166	0
**6**	1	N	N	227	12
**8**	1	Y	N	259	3
**9**	1	Y	Y	126	2
**10**	1	Y	N	149	114
**15**	1	Y	N	196	1
**16**	1	Y	Y	185	1
**20**	1	Y	Y	150	9
**21**	1	Y	N	133	0
**31**	1	Y	N	108	0
**32**	1	Y	N	143	2
**35**	1	y	N	236	19
**46**	1	Y	N	103	55
**47**	1	Y	Y	128	3
**48**	1	N	N	170	0
**50**	1	N	Y	142	1
**52**	1	Y	Y	123	0
**1**	1	N	Y	211.6	0
**2**	1	N	N	140.4	.
**5**	1	Y	Y	168	0
**7**	1	N	Y	149.6	.
**11**	1	Y	N	134.8	12
**12**	1	N	N	178	0
**13**	1	N	N	124	107
**14**	1	N	Y	119	0
**17**	1	N	Y	228.4	0
**18**	1	N	Y	192.4	4
**22**	1	N	Y	124	0
**25**	1	N	Y	172.4	0
**26**	1	Y	N	168	61
**27**	1	Y	Y	140.2	0
**36**	1	Y	Y	123.6	3
**37**	1	Y	N	112	93
**38**	1	N	N	100.8	0
**40**	1	N	N	160.4	0

**41**	1	Y	N	-	0
**43**	1	Y	N	144.8	0
**44**	1	N	N	125.8	0
**45**	1	Y	Y	141.2	0
**4**	2	N	Y	167	0
**6**	2	Y	N	174	74
**8**	2	Y	Y	192	0
**9**	2	N	Y	137	0
**10**	2	Y	Y	160	17
**15**	2	N	Y	137	2
**16**	2	Y	N	149	4
**20**	2	N	Y	193	2
**21**	2	N	N	145	19
**31**	2	Y	Y	120	2
**32**	2	N	N	129	8
**35**	2	N	N	160	6
**46**	2	Y	Y	78	8
**47**	2	N	N	152	0
**48**	2	N	Y	126	0
**50**	2	Y	Y	117	0
**52**	2	N	N	109	11
**1**	2	Y	Y	126	0
**2**	2	Y	Y	140	0
**5**	2	N	N	133.2	0
**7**	2	Y	N	146.8	2
**11**	2	N	N	121.2	21
**12**	2	Y	N	109.2	0
**13**	2	Y	Y	124.4	24
**14**	2	N	N	124.4	0
**17**	2	Y	Y	136	0
**18**	2	N	N	137.4	.
**22**	2	Y	Y	150.5	0
**25**	2	Y	N	154.8	0
**26**	2	N	Y	174	9
**27**	2	Y	N	125.6	0
**36**	2	Y	N	160.4	1
**37**	2	Y	Y	132.4	60
**38**	2	Y	N	100	0
**40**	2	Y	N	160.8	.

**41**	2	Y	Y	94	0
**43**	2	N	Y	130.4	.
**44**	2	Y	N	111.4	0
**45**	2	Y	N	110.4	0
**4**	3	N	N	206	6
**6**	3	Y	Y	248	24
**8**	3	N	N	195	0
**9**	3	N	N	129	1
**10**	3	N	Y	129	14
**15**	3	Y	Y	169	0
**16**	3	N	Y	165	0
**20**	3	Y	N	169	2
**21**	3	Y	Y	112	3
**31**	3	N	N	135	0
**32**	3	Y	Y	278	8
**35**	3	Y	Y	210	0
**46**	3	N	Y	72	23
**47**	3	Y	N	83	2
**48**	3	Y	Y	191	0
**50**	3	Y	N	156	31
**52**	3	Y	N	128	2
**1**	3	N	N	146	55
**2**	3	N	Y	162.8	0
**5**	3	N	Y	135.2	0
**7**	3	N	N	163	0
**11**	3	Y	Y	127	24
**12**	3	Y	Y	114.8	0
**13**	3	N	Y	128.8	.
**14**	3	Y	N	123	0
**17**	3	Y	N	150	0
**18**	3	Y	N	124.8	1
**22**	3	N	N	134.8	0
**25**	3	N	N	118.4	0
**26**	3	Y	Y	168.8	.
**27**	3	N	Y	124.4	4
**36**	3	N	N	109.6	0
**37**	3	N	Y	173.6	52
**38**	3	N	Y	116.4	0
**40**	3	Y	Y	143.6	.

**41**	3	Y	Y	129.2	0
**43**	3	N	N	150	0
**44**	3	N	Y	163.2	0
**45**	3	N	Y	139.4	0
**4**	4	Y	N	219	15
**6**	4	N	Y	163	8
**8**	4	N	Y	169	0
**9**	4	Y	N	148	4
**10**	4	N	N	111	3
**15**	4	N	N	141	0
**16**	4	N	N	191	10
**20**	4	N	N	147	5
**21**	4	N	Y	101	0
**31**	4	N	Y	127	0
**32**	4	N	Y	147	1
**35**	4	N	Y	170	0
**46**	4	N	N	83	20
**47**	4	N	Y	158	1
**48**	4	Y	N	180	0
**50**	4	N	N	106	9
**52**	4	N	Y	105	1
**1**	4	Y	N	168.4	128
**2**	4	Y	N	154.8	0
**5**	4	Y	N	154.2	2
**7**	4	Y	Y	137.4	0
**11**	4	N	Y	119.4	76
**12**	4	N	Y	135	2
**13**	4	Y	N	116.4	22
**14**	4	Y	Y	133	1
**17**	4	N	N	157.8	0
**18**	4	Y	Y	132.8	2
**22**	4	Y	N	123.6	0
**25**	4	Y	Y	127.6	0
**26**	4	N	N	172.8	2
**27**	4	N	N	113.6	4
**36**	4	N	Y	120.4	0
**37**	4	N	N	124.8	.
**38**	4	Y	Y	100.6	0
**40**	4	N	Y	177.8	0

**41**	4	N	N	132.8	0
**43**	4	Y	Y	145.4	0
**44**	4	Y	Y	107.8	0
**45**	4	N	N	120.8	0
