# Supplementary material for: Effects of Waiting Room and Feline Facial Pheromone Experience on Blood Pressure in Cats
Source: Front Vet Sci. 2021 Mar 5;8:640751. doi: 10.3389/fvets.2021.640751 (PMC7973014; doi:10.3389/fvets.2021.640751)
Supplement: Supplementary file 2 [file Data_Sheet_2.PDF]

Appendix 2: Demographic data and year evaluated for all cats. NM=neutered male; SF=spayed female.

Cat ID	Age (yr)	Sex	Weight (lbs)	Year evaluated
**4**	7	NM	15.7	2017
**6**	2	NM	18.3	2017
**8**	7	NM	14.7	2017
**9**	7	SF	15.9	2017
**10**	5	SF	12.5	2017
**15**	1	NM	7.1	2017
**16**	3	NM	12.3	2017
**20**	3	SF	10.3	2017
**21**	2	SF	8.9	2017
**31**	8	SF	13.6	2017
**32**	7	NM	18.4	2017
**35**	4	NM	12.4	2017
**46**	11	SF	8.7	2017
**47**	11	NM	17.1	2017
**48**	11	NM	9.3	2017
**50**	3	SF	9.7	2017
**52**	7	SF	12.3	2017
**1**	2	NM	8.25	2016
**2**	15	SF	7.7	2016
**5**	7	SF	8.75	2016
**7**	10	SF	9.8	2016
**11**	8	SF	11	2016
**12**	4	SF	12	2016
**13**	2	SF	7.5	2016
**14**	3	SF	12	2016
**17**	4	NM	9	2016
**18**	4	NM	14.5	2016
**22**	1	NM	12.5	2016
**25**	6	SF	8.75	2016
**26**	13	SF	10.5	2016
**27**	9	NM	12.75	2016
**36**	1	SF	6.5	2016
**37**	.	SF	8.5	2016
**38**	3	NM	7.25	2016

**40**	8	SF	7.75	2016
**41**	9	SF	8.3	2016
**43**	1	SF	7	2016
**44**	4	NM	12.5	2016
**45**	3	NM	16	2016
**4**	7	NM	15.7	2017
**6**	2	NM	18.3	2017
**8**	7	NM	14.7	2017
**9**	7	SF	15.9	2017
**10**	5	SF	12.5	2017
**15**	1	NM	7.1	2017
**16**	3	NM	12.3	2017
**20**	3	SF	10.3	2017
**21**	2	SF	8.9	2017
**31**	8	SF	13.6	2017
**32**	7	NM	18.4	2017
**35**	4	NM	12.4	2017
**46**	11	SF	8.7	2017
**47**	11	NM	17.1	2017
**48**	11	NM	9.3	2017
**50**	3	SF	9.7	2017
**52**	7	SF	12.3	2017
**1**	2	NM	8	2016
**2**	15	SF	7.5	2016
**5**	7	SF	8.75	2016
**7**	10	SF	10	2016
**11**	8	SF	10.7	2016
**12**	4	SF	11.5	2016
**13**	2	SF	7.5	2016
**14**	3	SF	11.5	2016
**17**	4	NM	9	2016
**18**	4	NM	14.5	2016
**22**	1	NM	12	2016
**25**	6	SF	9.5	2016
**26**	13	SF	9.8	2016
**27**	9	NM	11.5	2016
**36**	1	SF	6.8	2016
**37**		SF	8	2016
**38**	3	NM	7.2	2016

**40**	8	SF	7.8	2016
**41**	9	SF	7.75	2016
**43**	1	SF	.	2016
**44**	4	NM	12.25	2016
**45**	3	NM	16	2016
**4**	7	NM	15.7	2017
**6**	2	NM	18.3	2017
**8**	7	NM	14.7	2017
**9**	7	SF	15.9	2017
**10**	5	SF	12.5	2017
**15**	1	NM	7.1	2017
**16**	3	NM	12.3	2017
**20**	3	SF	10.3	2017
**21**	2	SF	8.9	2017
**31**	8	SF	13.6	2017
**32**	7	NM	18.4	2017
**35**	4	NM	12.4	2017
**46**	11	SF	8.7	2017
**47**	11	NM	17.1	2017
**48**	11	NM	9.3	2017
**50**	3	SF	9.7	2017
**52**	7	SF	12.3	2017
**1**	2	NM	8.5	2016
**2**	15	SF	8.5	2016
**5**	7	SF	8.5	2016
**7**	10	SF	9.9	2016
**11**	8	SF	11.5	2016
**12**	4	SF	11.25	2016
**13**	2	SF	7.5	2016
**14**	3	SF	12	2016
**17**	4	NM	9	2016
**18**	4	NM	14.75	2016
**22**	1	NM	12.25	2016
**25**	6	SF	8.75	2016
**26**	13	SF	10	2016
**27**	9	NM	12	2016
**36**	1	SF	6.7	2016
**37**	.	SF	7.75	2016
**38**	3	NM	7.25	2016

**40**	8	SF	7.75	2016
**41**	9	SF	8.25	2016
**43**	1	SF	7	2016
**44**	4	NM	12	2016
**45**	3	NM	16.5	2016
**4**	7	MN	15.7	2017
**6**	2	MN	18.3	2017
**8**	7	MN	14.7	2017
**9**	7	SF	15.9	2017
**10**	5	SF	12.5	2017
**15**	1	MN	7.1	2017
**16**	3	MN	12.3	2017
**20**	3	SF	10.3	2017
**21**	2	SF	8.9	2017
**31**	8	SF	13.6	2017
**32**	7	MN	18.4	2017
**35**	4	MN	12.4	2017
**46**	11	SF	8.7	2017
**47**	11	MN	17.1	2017
**48**	11	MN	9.3	2017
**50**	3	SF	9.7	2017
**52**	7	SF	12.3	2017
**1**	2	NM	8	2016
**2**	15	SF	7.25	2016
**5**	7	SF	8.5	2016
**7**	10	SF	10.25	2016
**11**	8	SF	10.75	2016
**12**	4	SF	11.25	2016
**13**	2	SF	7.75	2016
**14**	3	SF	12	2016
**17**	4	NM	9.5	2016
**18**	4	NM	15.3	2016
**22**	1	NM	12.75	2016
**25**	6	SF	9.5	2016
**26**	13	SF	10.5	2016
**27**	9	NM	11.75	2016
**36**	1	SF	6.9	2016
**37**	.	SF	7.8	2016
**38**	3	NM	8.25	2016

**40**	8	SF	8	2016
**41**	9	SF	8.5	2016
**43**	1	SF	7.75	2016
**44**	4	NM	12.75	2016
**45**	3	NM	17	2016
